# Prospective, Multicenter, Observational Study to Evaluate a Cell-Impermeable Endoprosthesis for Treatment of Stenosis or Occlusion within the Dialysis Outflow Circuit of an Arteriovenous (AV) Fistula or AV Graft (The WRAP Registry)

**DOI:** 10.1007/s00270-023-03531-w

**Published:** 2023-08-17

**Authors:** Dheeraj K. Rajan, Panagiotis M. Kitrou

**Affiliations:** 1 Department of Medical Imaging, University Medical Imaging Toronto/University of Toronto, University Health Network, 585 University Avenue, 1-PMB-287, Toronto, ON M5G 2N2 Canada; 2 Interventional Radiology, Patras University Hospital, Patras, Greece

**Keywords:** Hemodialysis, Patency, Occlusion, Stenosis, WRAPSODY Cell-Impermeable Endoprosthesis

## Abstract

**Purpose:**

Dysfunctional vascular access due to stenosis/occlusion within the arteriovenous fistula or graft (AVF/AVG) negatively affects the clinical management of patients undergoing hemodialysis. Results from the feasibility study of the WRAPSODY™ Cell-Impermeable Endoprosthesis demonstrated that the device can achieve high patency rates and maintain integrity of the dialysis outflow circuit. This study was designed to assess real-world evidence of safety and efficacy outcomes following device placement.

**Materials and Methods:**

This is a prospective, multicenter, non-investigational, post-market observational study of 500 patients at up to 50 centers worldwide with a mature AVF/AVG dialysis access (≥ 1 hemodialysis session) who experience stenosis/occlusion of the outflow circuit prior to placement of WRAPSODY. Patients will be divided into the following two cohorts: peripheral or central thoracic. Primary outcome measures include target lesion primary patency (TLPP) at 6 months and procedure and/or device-related events through 30 days post-procedure. Secondary outcome measures include TLPP, safety events, and the number of interventions needed to maintain patency through the 24 month study period. Exploratory endpoints include time to access abandonment, resumption of successful dialysis, functional patency, and pending available imaging data, any incidence of stent fractures, migration, or edge stenosis. Study enrollment began in June 2022, the last patient visit is expected in 2026.

**Discussion:**

It is expected that this study will provide real-world evidence regarding the performance of the WRAPSODY device in a diverse population of patients, which may encourage its use in the continuum of hemodialysis access management.

**Trial Registration:**

NCT05062291

**Graphical Abstract:**

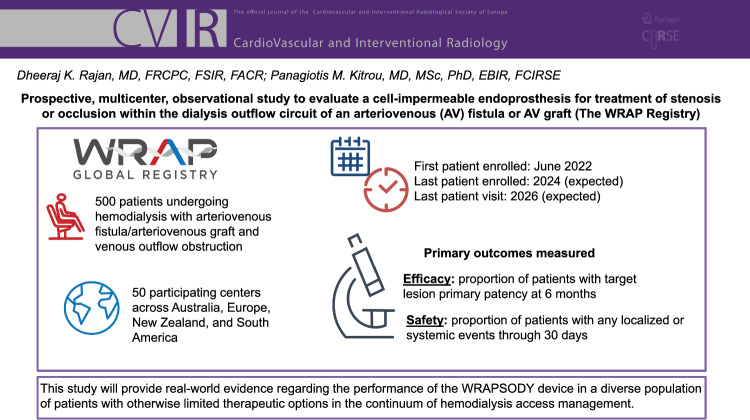

## Introduction

Hemodialysis is the most common form of kidney replacement therapy, accounting for nearly 90% of all dialysis methods [1, 2]. The success of hemodialysis requires continuous function of a vascular access that is traditionally accomplished through the creation of an arteriovenous fistula (AVF) or implanting an arteriovenous graft (AVG) [3, 4]. However, complications such as stenosis and/or occlusion are a leading cause of failure in the AVF and AVG, which can interfere with the ability of patients to undergo hemodialysis, resulting in potentially increased morbidity and life-threatening consequences [4–6].

Treatment options to adequately manage dysfunctional vascular access due to stenosis/occlusion are associated with limited patency. Surgery may assist with achieving extended patency of the access circuit, however, high rates of morbidity due to trauma preclude it from becoming a commonly employed treatment strategy and is instead reserved for non-salvageable vascular accesses [7–9]. A common endovascular alternative for the clinical management of venous stenosis/occlusion includes use of percutaneous transluminal angioplasty (PTA) [7, 10–14]. However, patency of PTA can vary widely, with average reported cumulative primary patency rates of 61% and 47% at 6 and 12 months, respectively [15], which often requires re-intervention(s) to maintain patency. Within the past 30 years, covered stents have emerged as an alternative to PTA to maintain patency of the hemodialysis access circuit [16]. The proposed advantages of covered stents over PTA are their stable and relatively inert matrix to support endothelialization coupled with their structural rigidity to help maintain patency of the circuit [17]. However, even with early covered stents, patency rates have been reported to decline over time with one study reporting approximately 70% of patients requiring re-intervention within one year of placement [18]. As a result, there has been an impetus to continue to innovate covered stents to improve patency of vascular access for hemodialysis.

The WRAPSODY™ Cell-Impermeable Endoprosthesis, a novel covered stent, was developed to help improve long-term vascular access. The device is a self-expanding endoprosthesis composed of a single nitinol wire encased between layers of fluoropolymer that helps to reduce stenosis and thrombus formation. A preclinical study that examined rates of stenosis following placement of the WRAPSODY device versus a conventional covered stent found that the device was associated with significantly lower rates of stenosis (2.1% vs. 10.3%, *p* = 0.009) [19]. Findings from the first-in-man study of the WRAPSODY device, that included 46 patients with access circuit stenosis treated with the device, demonstrated primary patency rates of 97.7% and 84.6% at 6 and 12 months, respectively, [20]. Based on the results of the first-in-man study in 2020, the WRAPSODY device received CE Mark approval.

Despite the promising efficacy outcomes associated with the WRAPSODY device [19, 20], there is an unmet need for real-world data describing clinical outcomes associated with the device, particularly in a large patient population. To help address this need, we describe the design of a prospective, multicenter, post-market, observational study (the WRAP Registry) that will document real-world data for safety and efficacy outcomes following use of the WRAPSODY device in 500 patients.

## Methods

### Study Design

The WRAP Registry is a prospective, multicenter, non-investigational, non-randomized, post-market, observational study conducted at up to 50 centers throughout Australia, Europe, New Zealand, and South America.

### Compliance with Ethical Standards

This study complies with the 1964 Helsinki Declaration and its later amendments. Ethics approval has been granted by the local ethics committees for each center participating in the study.

### Inclusion and Exclusion Criteria

The full inclusion and exclusion criteria are provided in Table 1. As this study seeks to describe the safety and efficacy outcomes associated with the use of the WRAPSODY device in a real-world population, the eligibility criteria are intentionally minimal to help capture the full range of clinical profiles of the study population and avoid bias for, or against, specific subgroups (e.g., disease states, anatomies).

**Table 1:** Full inclusion and exclusion criteria

Inclusion criteria	Exclusion criteria
Patient provides written informed consent for study participation	Patient has a planned surgical revision of access site
Patient is male or female, with an age ≥ 18 years at date of enrollment	Patient has a known or suspected infection of the hemodialysis access site, systemic infection, and/or septicemia
Patient is willing to comply with site standard of care procedures and follow-up visit schedules over 24 months	Patient has an uncorrectable coagulation disorder
Patient is undergoing chronic hemodialysis using the hemodialysis access that the intervention will be performed upon	Known hypersensitivity to nickel titanium alloy
The dialysis access is considered mature and has been used to deliver hemodialysis treatments for at least one session	Patient's hemodialysis access is anticipated to be abandoned within 6 months
Patient has stenosis or occlusion within the dialysis outflow circuit of an AV fistula or AV graft and is treated with the WRAPSODY Cell-Impermeable Endoprosthesis System in accordance with device Instructions for Use	Patient is scheduled for kidney transplant or peritoneal dialysis within the next 6 months post-procedure
	Full expansion of a PTA balloon cannot be achieved during predilatation
	Device would be placed in the superior vena cava
	Any inflow or outflow lesion that could jeopardize patency access long-term beyond the target treatment area

AV, arteriovenous; PTA, percutaneous transluminal angioplasty

### Study Cohort

Up to 500 adults, across participating centers, with stenosis or occlusion within the dialysis outflow circuit who meet the inclusion and exclusion criteria (Table 1) will be classified into one of two cohorts based on location of stenosis/occlusion: peripheral cases in both AVG and AVF or thoracic central cases in both AVG and AVF. Thoracic central cases will include the subclavian vein, brachiocephalic/innominate vein, and superior vena cava. Peripheral cases will include veins distal to the axillary vein at the junction of the entry of the cephalic vein (cephalic arch).

### Study Procedures

#### Screening and Enrollment

All consecutive patients who meet the eligibility criteria will be considered for enrollment in the study and provided additional information on the study, including informed consent.

All patients who are treated with the WRAPSODY device will be considered enrolled in the study. If the device is inserted into vasculature and treatment is attempted but the procedure is terminated without delivery of the covered stent, the only data collected will be device failure, malfunction details, and/or device-related adverse events. These patients will be followed through discharge for safety events only and exited from the study.

#### Intervention

All procedures will be conducted in accordance with each center’s standard of care and patient needs. There are no prohibited concomitant treatments. Deployment of WRAPSODY will be carried out in accordance with the Instructions for Use.

#### Data Monitoring

Safety events will be classified according to the International Organization for Standardization (ISO 14155:2020) definitions. An independent physician adjudicator will review and adjudicate safety events.

### Follow-up

Patients will be followed for up to 24 months. During the 24 month study period, data will be collected at the following timepoints post-procedure: 30 days, 6 months, 12 months, and 24 months. Data collected will include, as available, the following: physical assessment of vascular access circuit, dialysis status, any subsequent intervention(s) on the hemodialysis access circuit, device/procedure-related adverse events (up to 6 months only), and serious device or procedure-related adverse events, including death (Table 2). Patients unable to be assessed in-person at any of the designated timepoints for data collection will be contacted by phone to confirm their status. Upon completion of the study, all patients will be treated according to routine operating practices at the institution of their hemodialysis provider.

**Table 2:** Data collection schedule

Assessment	Baseline ≤ 30 Days	Index procedure (Enrollment)	Follow-up assessment 30 days (± 15 days) 6 months (180 ± 30 days) 12 months (360 ± 45 days) 24 months (720 ± 75 days)
Informed consent	X^a^		
Demographics	X		
Medical history	X		
Assessment of fistula or graft/access circuit	X	X	X
Dialysis status	X		X
Index procedure information^b^		X	
Subsequent interventions to access circuit			X
Device and/or procedure related serious adverse events^c^		X	X

^a^ Informed consent will be obtained for all patients prior to index procedure, or as soon as possible after the procedure but no later than time of patient discharge. Informed consent must be collected prior to any study data collection

^b^ The following data will be collected at index procedure through to discharge: lesion pre-treatment (e.g., percutaneous transluminal angioplasty, drug-eluting balloon therapy, thrombectomy); secondary (non-target) lesions (number and treatment) not treated with the WRAPSODY Cell-Impermeable Endoprosthesis; baseline angiographic assessments (e.g., reference vessel diameter, lesion length, predilatation), note: angiographic imaging of procedure will be retained for the duration of the study in case it is required for event adjudication by an independent physician adjudicator; location of stent graft placement; the WRAPSODY Cell-Impermeable Endoprosthesis details (e.g., number, size, and lot numbers); post-treatment of lesion/vessel; assessment of post-stent graft implantation lumen patency via angiogram at the conclusion of the index procedure; evaluation of total procedure time; identification of technical difficulties; adverse event observation, evaluation, and treatment; date of discharge

^c^ All potential device- and/or procedure-related adverse events and all deaths will be collected to 6 months. From 6 months, only serious and device- and/or procedure-related adverse events and all deaths will be collected

Patients may withdraw from the study at any time without prejudice. Reasons for early termination will be documented and data will be evaluated until the time of any patient’s withdrawal. Any patient(s) that withdraws from the study, or is/are lost to follow-up, will not be replaced.

Enrollment in this study began in June 2022; the last patient is expected to be enrolled in 2024 and the last patient visit is expected in 2026.

### Primary Outcome Measures

The primary outcome measures focus on the efficacy and safety of the device and generally align with standardized definitions reported previously [21, 22].

The primary efficacy outcome measure is the proportion of patients with target lesion primary patency (TLPP) at 6 months. TLPP is defined as freedom from clinically driven target lesion revascularization or target lesion thrombosis measured through 6 months post-procedure, which is the time interval of uninterrupted patency following the study procedure to the next intervention performed on the target lesion, or uncorrectable target lesion occlusion.

The primary safety outcome measure is the proportion of patients without any localized or systemic safety events through 30 days post-procedure that affect the access or venous outflow circuit and result in reintervention, hospitalization, or death (not including stenosis or thrombosis).

### Additional Outcome Measures Assessed

Secondary outcomes will include the proportion of patients with: TLPP at 12 and 24 months; assisted TLPP at 6, 12, and 24 months (defined as the time to loss of assisted primary patency of the target lesion, which is the time from the study procedure to uncorrectable target occlusion); access circuit primary patency at 6, 12, and 24 months (defined as time to loss of primary patency); post-procedure secondary patency outcomes at 6, 12, and 24 months (defined as the interval post-procedure until access circuit abandonment). Additional secondary outcome measures include serious adverse events across the 24 month study period, number of device observations, number of interventions to maintain access circuit patency, number of target lesion re-interventions to maintain target lesion patency, as well as the percentage of patients with device success, procedural success, and anatomic success.

Pending data availability, additional outcome measures will include index of patency function at 6, 12, and 24 months (defined as the time from the initial study procedure to complete access abandonment divided by the number of venous outflow circuit re-interventions to maintain hemodialysis); clinical success (defined as resumption of successful dialysis through existing access for ≥ 1 session post-procedure); functional patency of the access (defined as the ability to support dialysis) at 6, 12, and 24 months. Provided follow-up imaging is collected as part of the standard of care, additional outcome measures will include the incidences of reported stent fractures, migration, and edge stenosis.

### Statistics

Data will be organized into two analytical sets: an intent-to-treat (ITT) analysis set and a per-protocol (PP) analysis set. The ITT analysis set will include all patients enrolled in the study and will be considered the primary analysis set for all safety and efficacy outcome measures. The PP analysis set will include all enrolled patients who meet all inclusion and exclusion criteria with no major protocol deviations.

All analyses will be descriptive; no hypothesis testing is planned. Continuous variables will be summarized using mean, standard deviation, median, minimum, and maximum. Categorical variables will be summarized using frequencies and percentages will be reported with their associated confidence intervals. Time-to-event data will be summarized using Kaplan–Meier curves. All data will be analyzed using SAS version 9.4, or later (SAS Institute Inc., Cary, NC).

The sample size of 500 was determined to be sufficient to accurately characterize use of WRAPSODY in real-world practice based on clinical consensus of the study’s principal investigators. An interim analysis is not currently planned.

#### Expected Gain of Knowledge

To date, all available data published on WRAPSODY has reported excellent patency rates [19, 20]. While the positive preliminary findings related to the device are promising, there is a need for additional evidence to confirm the clinical utility of the device. The WRAP Registry study is designed to address this knowledge gap by describing the safety and efficacy outcomes associated with the device in a large and diverse patient population. As this study encompasses multiple centers globally, the results are expected to provide broad insight regarding the utility of the device that will be highly informative for healthcare decision-makers.

As patients undergoing hemodialysis typically reflect a uniquely vulnerable population with high morbidity [23, 24], there is an on-going need to continue optimizing their clinical management and provide healthcare providers with safe and effective options to extend hemodialysis access. The results from this study are expected to inform ways to continue to improve patient care. Based on the early feasibility results [20], we anticipate favorable patency outcomes with WRAPSODY relative to other currently available covered stents. The prior animal model [19] of WRAPSODY would suggest that unique design features including the integral cell-impermeable layer and softened end-rows may contribute to these patency results.

## Data Availability

Not applicable.

## Abbreviations

AVF: Arteriovenous fistula
AVG: Arteriovenous graft
ITT: Intent-to-treat
PP: Per-protocol
PTA: Percutaneous transluminal angioplasty
TLPP: Target lesion primary patency

## Declarations

### Human and Animal Participants

All procedures performed in studies involving human participants were in accordance with the ethical standards of the institutional and/or national research committee and with the 1964 Helsinki Declaration and its later amendments or comparable ethical standards.
