# Correction to: Prospective, Multicenter, Observational Study to Evaluate a Cell-Impermeable Endoprosthesis for Treatment of Stenosis or Occlusion within the Dialysis Outflow Circuit of an Arteriovenous (AV) Fistula or AV Graft (The WRAP Registry)

**DOI:** 10.1007/s00270-023-03565-0

**Published:** 2023-10-11

**Authors:** Dheeraj K. Rajan, Panagiotis M. Kitrou

**Affiliations:** 1 Department of Medical Imaging, University Medical Imaging Toronto/University of Toronto, University Health Network, 585 University Avenue, 1-PMB-287, Toronto, ON M5G 2N2 Canada; 2 Interventional Radiology, Patras University Hospital, Patras, Greece

**Correction to: Cardiovasc Intervent Radiol (2023) 46:1285–1291** **https://doi.org/10.1007/s00270-023-03531-w**

The article “Prospective, Multicenter, Observational Study to Evaluate a Cell-Impermeable Endoprosthesis for Treatment of Stenosis or Occlusion within the Dialysis Outflow Circuit of an Arteriovenous (AV) Fistula or AV Graft (The WRAP Registry)”, written by Rajan, D. K. and Kitrou, P. M., was originally published Online First without Open Access. After publication in volume 46, issue 9, page 1285–1291 the author decided to opt for Open Choice and to make the article an Open Access publication. Therefore, the copyright of the article has been changed to © The Author(s) 2023 and the article is forthwith distributed under the terms of the Creative Commons Attribution 4.0 International License, which permits use, sharing, adaptation, distribution and reproduction in any medium or format, as long as you give appropriate credit to the original author(s) and the source, provide a link to the Creative Commons licence, and indicate if changes were made. The images or other third party material in this article are included in the article’s Creative Commons licence, unless indicated otherwise in a credit line to the material. If material is not included in the article’s Creative Commons licence and your intended use is not permitted by statutory regulation or exceeds the permitted use, you will need to obtain permission directly from the copyright holder. To view a copy of this licence, visit http://creativecommons.org/licenses/by/4.0

